# Association of Childhood and Midlife Neighborhood Socioeconomic Position With Cognitive Decline

**DOI:** 10.1001/jamanetworkopen.2023.27421

**Published:** 2023-08-04

**Authors:** Anna M. Kucharska-Newton, James Russell Pike, Jinyu Chen, Josef Coresh, A. Richey Sharret, Thomas Mosley, Priya Palta

**Affiliations:** 1 Department of Epidemiology, University of North Carolina at Chapel Hill; 2 Department of Epidemiology, University of Kentucky, Lexington; 3 Department of Epidemiology, Johns Hopkins University, Baltimore, Maryland; 4 Department of Neurology, University of Mississippi Medical Center, Jackson; 5 Department of Medicine, University of Mississippi Medical Center, Jackson; 6 Department of Neurology, University of North Carolina at Chapel Hill

## Abstract

**Importance:**

Early-life socioeconomic adversity may be associated with poor cognitive health over the life course.

**Objective:**

To examine the association of childhood and midlife neighborhood socioeconomic position (nSEP) with cognitive decline.

**Design, Setting, and Participants:**

This cohort study included 5711 men and women enrolled in the community-based Atherosclerosis Risk in Communities (ARIC) Study with repeated cognitive data measured over a median 27.0 years (IQR, 26.0-27.9 years) (1990-2019). Statistical analysis was performed from December 2022 through March 2023.

**Exposure:**

Residence addresses for ARIC Study cohort participants were obtained at midlife (1990-1993) and as recalled addresses at 10 years of age (childhood). A composite nSEP *z* score was created as a sum of *z* scores for US Census–based measures of median household income; median value of owner-occupied housing units; percentage of households receiving interest, dividend, or net rental income; percentage of adults with a high school degree; percentage of adults with a college degree; and percentage of adults in professional, managerial, or executive occupations. Childhood nSEP and midlife nSEP were modeled as continuous measures and discretized into tertiles.

**Main Outcomes and Measures:**

A factor score for global cognition was derived from a battery of cognitive tests administered at 5 in-person visits from baseline to 2019. The rate of cognitive decline from 50 to 90 years of age was calculated by fitting mixed-effects linear regression models with age as the time scale and adjusted for race, sex, birth decade, educational level, and presence of the apolipoprotein E ε4 allele.

**Results:**

Among 5711 ARIC Study participants (mean [SD] baseline age, 55.1 [4.7] years; 3372 women [59.0%]; and 1313 Black participants [23.0%]), the median rate of cognitive decline was −0.33 SDs (IQR, −0.49 to −0.20 SDs) per decade. In adjusted analyses, each 1-SD-higher childhood nSEP score was associated with a slower (β, −9.2%; 95% CI, −12.1% to −6.4%) rate of cognitive decline relative to the sample median. A comparable association was observed when comparing the highest tertile with the lowest tertile of childhood nSEP (β, −17.7%; 95% CI, −24.1% to −11.3%). Midlife nSEP was not associated with the rate of cognitive decline.

**Conclusions and Relevance:**

In this cohort study of contextual factors associated with cognitive decline, childhood nSEP was inversely associated with trajectories of cognitive function throughout adulthood.

## Key Points

**Question:**

Is neighborhood socioeconomic position (nSEP) associated with cognitive decline?

**Findings:**

In this longitudinal cohort study of 5711 men and women, the rate of cognitive decline from midlife to older adulthood decreased by 9.2% with each 1-SD increase in childhood nSEP. No associations of midlife nSEP with cognitive decline were observed.

**Meaning:**

This study suggests that early childhood experiences are associated with cognitive trajectories in adulthood.

## Teaser

This cohort study uses data from the Atherosclerosis Risk in Communities Study to examine the association of childhood and midlife neighborhood socioeconomic position with cognitive decline.

## Introduction

Early-life experiences are foundational to brain development and have strong implications for the trajectory of cognitive function throughout life.^1^ Such trajectories may be direct, through the effect of childhood life circumstances on brain development^2^ during sensitive cognitive developmental periods,^3^ or may be corollary to the accumulation of adverse vascular risk factors that begins in childhood.^4^ Early-life experiences are highly correlated with childhood socioeconomic position (SEP), which is often defined according to the educational level and/or occupation of the individual’s parents or as a contextual characteristic of the environment in which children are born and live.^5^ Beginning with early studies by Barker et al,^6,7^ which highlighted the importance of prenatal and early-childhood SEP to cardiovascular health in adulthood, there now exists extensive research documenting the risk of adverse cardiovascular outcomes in adulthood in association with childhood SEP that is not mitigated by adulthood SEP.^8,9^ Likewise, multiple studies provide evidence for a greater risk of low cognitive performance in adulthood and a subsequently greater likelihood of dementia at a younger age in association with low SEP in childhood.^10,11^ However, the few studies examining the association of childhood SEP with the rate of cognitive decline are conflicting.^10,12,13,14^ Moreover, most of those studies have operationalized childhood SEP exposure through the lens of parental educational level, occupation, and financial stability without consideration of the neighborhood context, which, when measured in adulthood, is known to be associated with cognitive function.^15,16,17^ In this investigation, we used life course SEP measures combined with repeated assessments of cognitive function available from the Atherosclerosis Risk in Communities (ARIC) Study cohort^18^ to examine the association of neighborhood SEP (nSEP), measured from childhood through midlife, with the rate of cognitive decline from midlife to older adulthood.

## Methods

### Study Population

Details describing the ARIC Study cohort have been published elsewhere.^18^ In brief, 15 792 men and women 45 to 64 years of age were recruited through population-based sampling conducted in 4 geographically distinct communities of Washington County, Maryland; Jackson, Mississippi; Forsyth County, North Carolina; and selected suburbs of Minneapolis, Minnesota. ARIC Study participants completed a clinic-based examination at visit 1 (1987-1989) and were reexamined at visit 2 (1990-1992), visit 3 (1993-1995), and visit 4 (1996-1998). The ARIC Neurocognitive Study (ARIC-NCS) was initiated during visit 5 (2011-2013) and continued during visit 6 (2016-2017) and visit 7 (2018-2019). The protocol for each visit was approved by the institutional review board of each participating field center (University of North Carolina at Chapel Hill, University of Mississippi Medical Center, Johns Hopkins University, University of Minnesota, and Wake Forest University), and oral informed consent was obtained at each visit. This cohort study followed the Strengthening the Reporting of Observational Studies in Epidemiology (STROBE) reporting guideline.^19^

### Exposure

Residence addresses for ARIC Study participants were obtained at visit 2 (1990-1993; midlife) and as recalled address in childhood (at 10 years of age) as part of the ARIC Lifecourse Socioeconomic Status Study conducted from 2002 to 2004.^20^ Geocoded addresses were used to place participants within geographically defined neighborhoods. A composite nSEP *z* score was calculated as a sum of *z* scores for US Census–based measures of (1) median household income; (2) median value of owner-occupied housing units; (3) percentage of households receiving interest, dividend, or net rental income; (4) percentage of adults with a high school degree; (5) percentage of adults with a college degree; and (6) percentage of adults in professional, managerial, or executive occupations.^21^ The childhood nSEP *z* score was derived from the 1930-1950 US Census data, which is available at only the county level. Midlife nSEP was based on US Census data available at the Census-tract level. Childhood nSEP and midlife nSEP were examined as continuous variables winsorized at the 1st and 99th percentiles and as tertiles of the overall distribution.

### Outcome

An in-person battery of neuropsychological tests was conducted in a quiet room by trained examiners using standardized protocols at visits 2, 4, 5, 6, and 7. During visits 2 and 4, the battery included the Word Fluency Test,^22^ Digit Symbol Substitution Test,^23^ and Delayed Word Recall Test.^24^ Beginning at visit 5, the battery additionally incorporated the Digit Span Backward Test,^23^ Boston Naming Test,^25^ Animal Naming Test,^22^ Trail Making Tests A and B,^26^ Incidental Learning Test,^27^ and Logical Memory Test.^23^ The tests available at each visit were used to compute a global cognition factor score at each visit.^28^

### Covariates

Sex, race, date of birth, and educational attainment were self-reported at visit 1. Date of birth was used to calculate the age in years at each visit. A categorical classification of race and center (hereafter referred to as race-center) was specified to account for the design of ARIC as well as possible systematic differences in health care resources and access associated with race and geography. Blood samples obtained during clinic visits were analyzed for apolipoprotein E (*APOE*) ε4 alleles using the TaqMan assay (Applied Biosystems).^29^ Additional information regarding study participants’ characteristics was ascertained at visit 2 and included high-density and low-density lipoprotein cholesterol level, body mass index, systolic blood pressure, hypertension, diabetes, multimorbidity, and physical activity (eMethods in Supplement 1).

### Statistical Analysis

Statistical analysis was performed from December 2022 through March 2023. The analytic sample (eFigure in Supplement 1) was restricted to participants enrolled in the ARIC-NCS (2011-2013; n = 6538), excluding those missing childhood nSEP (n = 795); those participants of race other than Black or White and, due to small sample sizes, Black participants in Maryland and Minnesota (n = 22); and those participants with an unknown level of educational attainment (n = 10). The baseline was defined as the first neuropsychological evaluation conducted at visit 2 (1990-1992). The median follow-up time was 27.0 years (IQR, 26.0-27.9 years) across a mean (SD) of 4.6 (0.7) neuropsychological evaluations.

To mitigate bias caused by informative attrition, multivariate imputation by chained equations with auxiliary variables was used to impute predeath cognitive factor scores among participants who did not complete a neuropsychological evaluation at a given visit (eMethods in Supplement 1).^30^ Ten imputed data sets were generated, exceeding the 6 imputations suggested by a quadratic formula.^31^ Imputed data sets were analyzed in a 2-stage process.

During the first stage, a linear mixed-effects model (LMM) estimated the rate of cognitive decline for each participant. The LMM specified time from 50 years of age as the time scale, used the maximum likelihood estimation, used an unstructured variance-covariance matrix, and incorporated a random intercept and slope. Sex and race-center were included as fixed effects, and an interaction with time was specified. The median rate of decline in the analytic sample was −0.33 SDs (IQR, −0.49 to −0.20 SDs) per decade.

In the second stage, a percentage difference was calculated as the rate of cognitive decline per participant divided by the median rate of decline in the analytic sample. Negative percentages represent slower cognitive decline, while positive percentages denote more rapid decline. Linear mixed-effects models with a random intercept at the neighborhood level were used to estimate the association between nSEP (continuous measures and discretized as tertiles) and the percentage difference from the median rate. Participant-specific rates of cognitive decline were also discretized into quintiles and analyzed in multinomial logistic mixed-effects models (MLMMs) that estimated the odds of belonging to a specific quintile relative to the third quintile. Specifically, we estimated the odds of having the least cognitive decline (fifth quintile) relative to the median (third quintile) or the most cognitive decline (first quintile) relative to the median (third quintile).

The childhood nSEP and midlife nSEP were examined in 2 models. Model 1 adjusted for sex and race-center. Model 2 additionally adjusted for birth decade, educational level, and presence of *APOE* ε4 alleles. All person-level covariates were group mean centered at the neighborhood level.^32^ The neighborhood-level mean values were reintroduced to accurately adjust for within- and between-neighborhood effects.^33^ An interaction between time and each covariate was specified. Parameter estimates from the analysis of imputed data were combined according to Rubin’s rules.^34^

Exploratory analyses examined moderation and effect modification by race, birth decade, sex, and multimorbidity in fully adjusted models. Two-sided *P* values for additive interactions in LMMs and multiplicative interactions in MLMMs were calculated by specifying a cross-level interaction between nSEP and a person-level covariate. *P* values for additive interactions in MLMMs were computed by calculating the relative excess risk due to interaction.^35^ Statistical significance was defined as *P* < .05. Point estimates and 95% CIs from stratified models were used to evaluate effect modification. Race-stratified restricted cubic spline models with knots placed at the 5th, 27.5th, 50th, 72.5th, and 95th percentiles assessed how the nonnormal distribution of nSEP altered the association with cognitive decline. All analyses were executed in SAS, version 9.4 (SAS Institute Inc), with the exception of the multiple imputation, which was performed in Stata, version 14.0 (StataCorp LLC).

## Results

At baseline, the mean (SD) age of the 5711 participants was 55.1 (4.7) years, 3372 participants (59.0%) were women, 2339 participants (41.0%) were men, and 1313 participants (23.0%) were Black (Table). The mean (SD) of the nSEP *z* score was 0.5 (4.0) at childhood and 1.1 (4.8) at midlife. Compared with participants in the lowest tertile of childhood nSEP, participants in the intermediate or highest tertiles were more likely to be younger and White, had more years of formal education and higher cognitive scores, and exhibited lower cardiometabolic risk. A similar, albeit smaller, gradient of participant characteristics was observed across the tertiles of midlife nSEP (eTable 1 in Supplement 1).

**Table.** Baseline Characteristics of the Study Population by Tertiles of nSEP During Childhood: The Atherosclerosis Risk in Communities Study, 1990-2019^a^

Characteristic	Participants, No. (%)				*P* value for trend^b^
	All (N = 5711)	Tertile of childhood nSEP, *z* score range			
		Low, −9.39 to −1.95 (n = 1906)	Intermediate, −1.93 to 2.16 (n = 1901)	High, 2.23 to 20.25 (n = 1904)	
Age, mean (SD), y	55.1 (4.7)	58.1 (4.2)	54.3 (4.4)	52.8 (3.8)	<.001
Decade of birth					
1920-1929	687 (12.0)	424 (22.2)	182 (9.6)	81 (4.3)	<.001
1930-1939	3355 (58.7)	1293 (67.8)	1087 (57.2)	975 (51.2)	
1940-1949	1669 (29.2)	189 (9.9)	632 (33.2)	848 (44.5)	
Sex					
Female	3372 (59.0)	1118 (58.7)	1139 (59.9)	1115 (58.6)	.95
Male	2339 (41.0)	788 (41.3)	762 (40.1)	789 (414)	
Black race	1313 (23.0)	581 (30.5)	325 (17.1)	407 (21.4)	<.001
Race and center					
White, Forsyth County, NC	1169 (20.5)	488 (25.6)	279 (14.7)	402 (21.1)	<.001
Black, Forsyth County, NC	84 (1.5)	33 (1.7)	14 (0.7)	37 (1.9)	
White, Minneapolis, MN	1699 (29.7)	205 (10.8)	557 (29.3)	937 (49.2)	
White, Washington County, MD	1530 (26.8)	632 (33.2)	740 (38.9)	158 (8.3)	
Black, Jackson, MS	1229 (21.5)	548 (28.8)	311 (16.4)	370 (19.4)	
Educational level					
Less than high school	827 (14.5)	436 (22.9)	236 (12.4)	155 (8.1)	<.001
High school, GED certification, or vocational school	2421 (42.4)	828 (43.4)	837 (44.0)	756 (39.7)	
College, graduate, or professional school	2463 (43.1)	642 (33.7)	828 (43.6)	993 (52.2)	
≥1 *APOE* ε4 allele, No./total No. (%)	1594/5527 (28.8)	512/1855 (27.6)	558/1826 (30.6)	524/1846 (28.4)	.60
Systolic blood pressure					
No.	5679	1893	1887	1899	NA
Mean (SD), mm Hg	117.5 (16.6)	119.7 (17.1)	116.3 (16.0)	116.3 (16.4)	<.001
Hypertension	1783 (31.2)	715 (37.5)	533 (28.0)	535 (28.1)	<.001
BMI					
No.	5677	1893	1886	1898	NA
Mean (SD)	27.7 (5.1)	27.8 (5.2)	27.7 (5.0)	27.6 (5.2)	.34
Diabetes, No/total No. (%)	497/5660 (8.8)	185/1885 (9.8)	160/1882 (8.5)	152/1893 (8.0)	.05
Sports participation					
No.	5708	1904	1901	1903	NA
Mean (SD), min/wk	668.1 (807.4)	598.3 (766.2)	675.0 (814.3)	730.9 (835.1)	<.001
HDL cholesterol					
No.	5644	1877	1879	1888	NA
Mean (SD), mg/dL	50.9 (16.7)	50.2 (15.9)	50.8 (17.3)	51.7 (17.0)	.02
LDL cholesterol					
No.	5576	1859	1855	1862	NA
Mean (SD), mg/dL	130.8 (35.0)	132.9 (33.8)	131.5 (35.8)	127.9 (35.1)	<.001
Multimorbidity	473 (8.3)	205 (10.8)	129 (6.8)	139 (7.3)	<.001
Childhood nSEP					
No.	5711	1906	1901	1904	NA
Mean (SD)	0.5 (4.0)	−3.7 (1.6)	0.4 (1.2)	4.9 (2.8)	<.001
Midlife nSEP					
No.	5538	1802	1861	1875	NA
Mean (SD)	1.1 (4.8)	−0.2 (4.7)	1.1 (4.6)	2.2 (4.8)	<.001
Global cognition, mean (SD)					
No.	5688	1897	1893	1898	NA
Mean (SD)	0.0 (1.0)	−0.3 (1.0)	0.1 (0.9)	0.2 (1.0)	<.001
Death by 2019	1326 (23.2)	574 (30.1)	414 (21.8)	338 (17.8)	<.001

Abbreviations: BMI, body mass index (calculated as weight in kilograms divided by height in meters squared); GED, General Educational Development; HDL, high-density lipoprotein; LDL, low-density lipoprotein; NA, not applicable; nSEP, neighborhood socioeconomic position.

SI conversion factors: To convert HDL cholesterol and LDL cholesterol to millimoles per liter, multiply by 0.0259.

^a^ Study baseline (1990-1992) defined as first year in which participant in Atherosclerosis Risk in Communities Study completed neuropsychological assessment.

^b^ Univariate baseline differences in study variables were assessed using linear regression, Cochran-Armitage trend tests, and Cochran-Mantel-Haenszel trend tests as appropriate.

### Rate of Cognitive Decline

**Figure 1.**
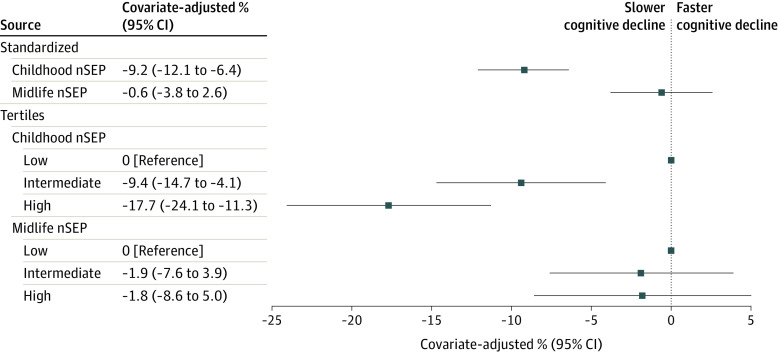
Estimates of Percentage Difference From Median Rate of Cognitive Decline Between 50 and 90 Years of Age by Neighborhood Socioeconomic Position (nSEP) During Childhood or Midlife: The Atherosclerosis Risk in Communities Study, 1990-2019 (N = 5711) A 2-stage process was used to estimate participant-specific cognitive decline between 50 and 90 years of age. Linear mixed-effects models with random intercepts at the neighborhood level estimated the association between nSEP and the percentage difference from the median rate of cognitive decline in the analytic sample. Separate models were fit for each measure of childhood and midlife nSEP. Each model was adjusted for sex, race and center, birth decade, education, and *APOE* ε4.

**Figure 2.**
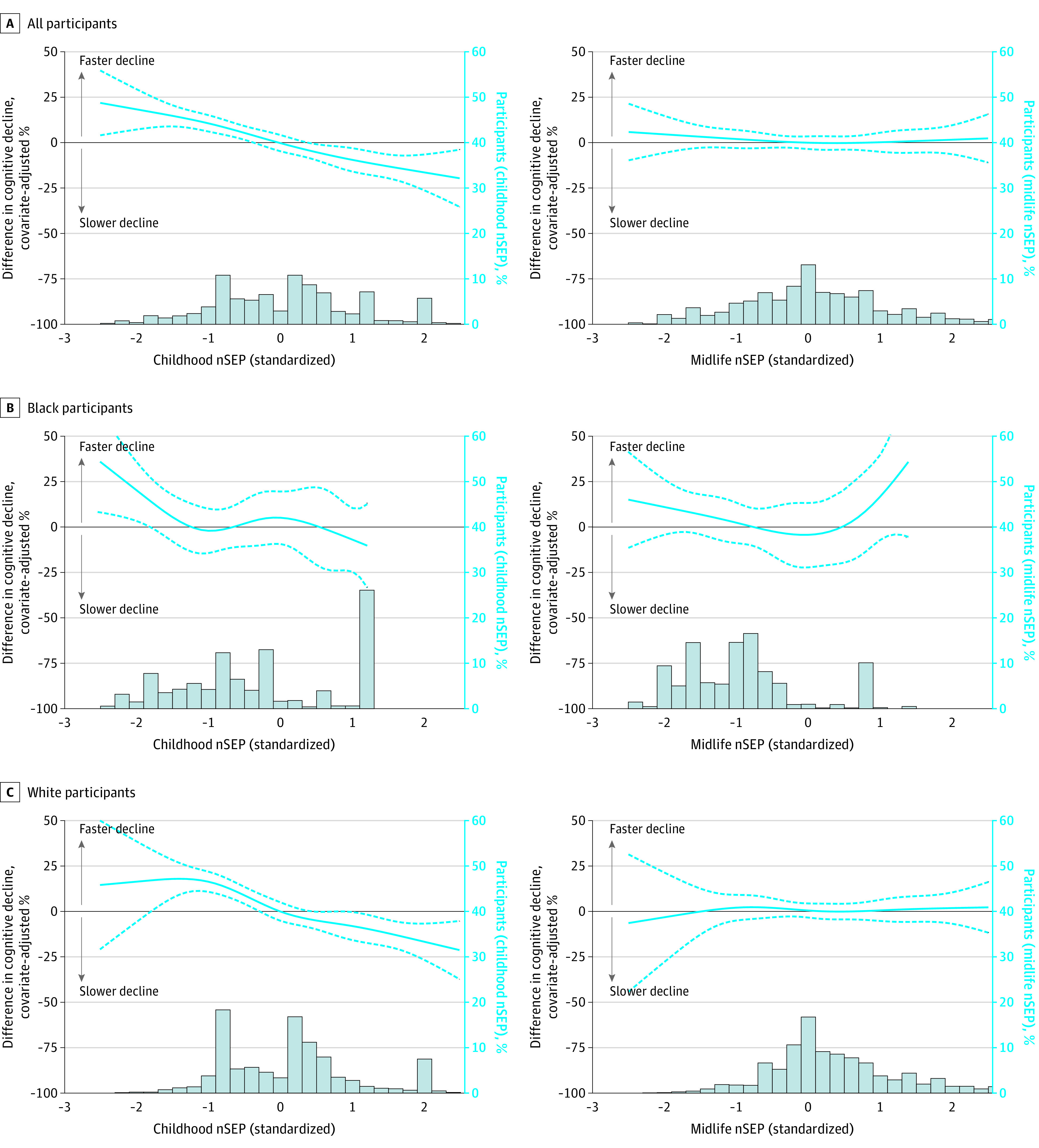
Histograms and Restricted Cubic Spline Model Estimates of the Percentage Difference From Median Rate of Cognitive Decline Between 50 and 90 Years of Age by Continuous Standardized Measures of Neighborhood Socioeconomic Position (nSEP) During Childhood or Midlife Stratified by Race: The Atherosclerosis Risk in Communities Study, 1990-2019 (N = 5711) A 2-stage process was used to estimate participant-specific cognitive decline between 50 and 90 years of age. Linear mixed-effects models with random intercepts at the neighborhood level and restricted cubic splines estimated the association between nSEP and the percentage difference from the median rate of cognitive decline in the analytic sample. Knots for the splines were placed at the 5th, 27.5th, 50th, 72.5th, and 95th percentiles. Separate models were fit for each measure of childhood and midlife nSEP. Effect modification was evaluated by stratifying the data set by race. The primary models were adjusted for sex, race and center, birth decade, education, and *APOE* ε4. Models fit to race-stratified data were adjusted for center instead of race and center. Histograms depict standardized nSEP during childhood or midlife. Solid lines indicate the point estimates, and dashed lines indicate the 95% CIs.

The median rate of cognitive decline among the cohort was −0.33 SDs (IQR, −0.49 to −0.20 SDs) per decade. In models adjusted for sex and race-center, a 1-SD-higher nSEP during childhood was associated with a slower (β, −22.6%; 95% CI, −25.4% to −19.9%) rate of cognitive decline relative to the sample median. After additionally adjusting for birth decade, educational level, and the presence of *APOE* ε4 alleles (Figure 1), the association was attenuated but remained statistically significant (β, −9.2%; 95% CI, −12.1% to −6.4%). A comparable association was observed when comparing the highest tertile with the lowest tertile of childhood nSEP (β, −17.7%; 95% CI, −24.1% to −11.3%). In contrast, the association of midlife nSEP was considerably smaller and not statistically significant in fully adjusted models. This pattern persisted when both exposures were included in the same model (eTable 2 in Supplement 1) and was further supported by restricted cubic spline models that depict a largely linear association (Figure 2).

### Quintiles of Cognitive Decline

**Figure 3.**
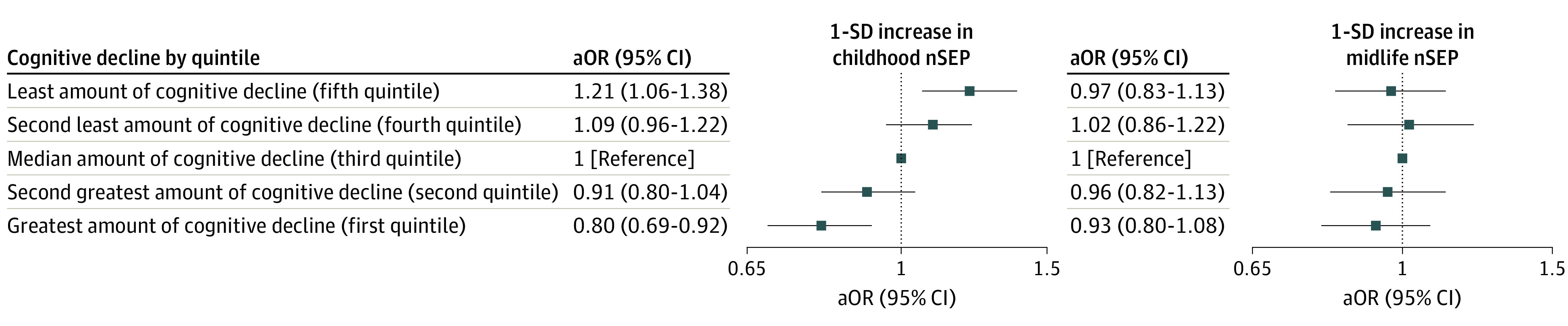
Estimates of the Odds of Belonging to a Specific Quintile of Cognitive Decline Between 50 and 90 Years of Age by Continuous Standardized Measures of Neighborhood Socioeconomic Position (nSEP) During Childhood or Midlife: The Atherosclerosis Risk in Communities Study, 1990-2019 (N = 5711) A 2-stage process was used to estimate participant-specific cognitive decline between 50 and 90 years of age. Multinomial logistic mixed-effects models with random intercepts at the neighborhood level estimated the association between nSEP and the odds of belonging to a specific quintile of cognitive decline. Separate models were fit for each measure of childhood and midlife nSEP. Each model was adjusted for sex, race and center, birth decade, education, and *APOE* ε4. aOR indicates covariate-adjusted odds ratio.

When cognitive decline was discretized into quintiles (Figure 3; eTables 3-5 in Supplement 1), individuals in the top quintile experienced a mean (SD) cognitive decline of −0.10 (0.09) SDs per decade. A 1-SD-higher childhood nSEP was associated with higher odds (odds ratio, 1.21; 95% CI, 1.06-1.38) of belonging to the top (fifth) quintile (ie, the least amount of cognitive decline) and lower odds (odds ratio, 0.80; 95% CI, 0.69-0.92) of belonging to the bottom (first) quintile (ie, the greatest amount of cognitive decline) relative to the median (third) quintile. This finding was replicated when examining tertiles of nSEP and when examining childhood and midlife nSEP in the same model.

### Exploratory Analyses

Exploratory analyses did not reveal consistent evidence of interactions (eTables 6 and 7 in Supplement 1) but did suggest that the protective association of higher childhood nSEP is greater among White participants who may also live in neighborhoods with a higher nSEP (childhood range, −9.3 to 20.2 SDs; midlife range, −8.6 to 14.8 SDs) than the maximum observed for Black participants (childhood range, −9.4 to 7.4 SDs; midlife range, −12.8 to 9.5 SDs).

## Discussion

In this large, well-characterized cohort of community-dwelling adults, we found that higher nSEP in childhood at 10 years of age was associated with protection against cognitive decline between 50 and 90 years of age. Although membership in the high tertile compared with low tertile of the childhood nSEP was associated with a 17.7% slower rate of cognitive decline, the protective association of childhood nSEP was not limited to those within the narrow highest childhood nSEP category. The quintile analysis permitted us to assess whether there is an outlying group with exceptional characteristics within the more favorable childhood nSEP categories, such as individuals in the top quintile, for whom the mean (SD) cognitive decline was −0.10 (0.09) SDs per decade. On the contrary, the monotonic continuum of the odds ratios examining the association of childhood nSEP with membership in quintiles of the rate of cognitive decline suggests only a stepwise incremental benefit of improvements in childhood nSEP with cognitive health in adulthood.

Despite the importance of childhood as a foundational period for determining health outcomes over the life course and the recognized association of SEP with cognitive development, investigations of the association of childhood SEP at the neighborhood level with person-level cognitive decline are sparse. Data from several cohorts are inconclusive^10,36,37,38,39^; however, a cross-cohort examination of the Whitehall II study,^40^ the Health and Retirement Study,^41^ and the Kame Project^42^ provides evidence for the association of poor individual-level SEP during childhood, defined as low parental educational attainment and financial hardships, with a faster rate of cognitive decline.^13^ Our focus on neighborhood-level SEP adds an important contextual understanding of how the early-life socioeconomic environment, beyond individual-level socioeconomic factors, contributes to cognitive health over the life course. This potentially modifiable social factor can serve as a target for interventions aimed at delaying the onset of cognitive impairment and decreasing the population burden of dementia.

Our findings fit well within the life course model,^43^ which postulates the existence of pathways linking early life experiences^44^ with adult health. Our observations also conform to the conceptual framework of formative periods of intense brain development during childhood and through adolescence and young adulthood that are highly sensitive to the environment.^45,46^ Adverse childhood socioeconomic conditions can serve as a marker of deprivation in cognitive exposures,^47^ which may be associated with adverse changes in synaptic development and associated cognitive functions.^48^ Low childhood SEP has been found to be associated with low hippocampal^49,50^ and amygdala^50^ volumes and with white matter hyperintensities in adulthood,^51^ suggesting that early socioeconomic adversity may be associated with distinct biological brain development pathways^52^ that are associated with accelerated cognitive decline.^53^

An important factor that remains unclear is whether the association of childhood socioeconomic adversity with brain function in adulthood is direct^54^ or mediated by midlife SEP.^38,39^ Results of our analyses did not provide evidence for an association of midlife nSEP with cognitive decline, suggesting that childhood nSEP may have a direct association with cognitive decline. Furthermore, the finding that, in our study, the association of childhood nSEP with cognitive decline was robust to adjustment for individual educational attainment and midlife nSEP also bolsters this conclusion. However, we cannot rule out a more nuanced multilevel process in which individual-level SEP, but not neighborhood-level SEP, in midlife, beyond educational attainment, mediates the association between childhood nSEP and cognitive decline.^55^

### Limitations

This study has some limitations. A key limitation was the lack of US Census information at the census-tract level during childhood, which may have obscured the observation of potential microgeographic variation in nSEP that may have attenuated the associations of childhood nSEP with cognitive decline relative to those observed for midlife nSEP. Informative attrition may or may not have introduced meaningful bias despite efforts to minimize bias using multiple imputation methods.^56^ Specifically, the observed median rate of cognitive decline was both slower^57,58^ and faster^59^ than the mean rate of decline reported in other studies, which may result in a misleading representation of the percentage difference because the magnitude is relative to the selected reference in our study population.

## Conclusions

In this cohort study, the observed gradient in the association of childhood nSEP with the rate of cognitive decline has implications for health and social policy interventions that can benefit children during early developmental stages and promote the closing of the persistent socioeconomic gap in cognitive outcomes at critical developmental periods. Place-based interventions aimed at reducing neighborhood poverty and improving social cohesion provide promising examples.^60^

## Notes

Supplement 1.
eMethods.
**eTable 1.** Baseline Characteristics of the Study Population by Tertiles of Neighborhood Socioeconomic Position During Midlife: The ARIC Study 1990-2019 (N=5538) **eTable 2.** Combined Estimates of Percentage Difference From Median Rate of Cognitive Decline Between the Ages of 50 and 90 Years by Neighborhood Socioeconomic Position During Childhood and Midlife: The ARIC Study 1990-2019 (N=5711) **eTable 3.** Estimates of the Odds of Belonging to a Specific Quintile of Cognitive Decline Between the Ages of 50 and 90 Years by Tertiles of Neighborhood Socioeconomic Position During Childhood or Midlife: The ARIC Study 1990-2019 (N=5711) **eTable 4.** Combined Estimates of the Odds of Belonging to a Specific Quintile of Cognitive Decline Between the Ages of 50 and 90 Years by Continuous, Standardized Measures of Neighborhood Socioeconomic Position During Childhood and Midlife: The ARIC Study 1990-2019 (N=5711) **eTable 5.** Combined Estimates of the Odds of Belonging to a Specific Quintile of Cognitive Decline Between the Ages of 50 and 90 Years by Tertiles of Neighborhood Socioeconomic Position During Childhood and Midlife: The ARIC Study 1990-2019 (N=5711) **eTable 6.** Stratified Estimates of Percentage Difference From Median Rate of Cognitive Decline Between the Ages of 50 and 90 Years by Continuous, Standardized Measures of Neighborhood Socioeconomic Position During Childhood or Midlife: The ARIC Study 1990-2019 (N=5711) **eTable 7.** Stratified Estimates of the Odds of Belonging to a Specific Quintile of Cognitive Decline Between the Ages of 50 and 90 Years by Continuous, Standardized Measures of Neighborhood Socioeconomic Position During Midlife: The ARIC Study 1990-2019 (N=5711) **eFigure.** Flowchart of Participants Selected for Analysis
eReferences.

Supplement 2.
Data Sharing Statement
