# Supplementary material for: Association of Childhood and Midlife Neighborhood Socioeconomic Position With Cognitive Decline
Source: JAMA Netw Open. 2023 Aug 4;6(8):e2327421. doi: 10.1001/jamanetworkopen.2023.27421 (PMC10403777; doi:10.1001/jamanetworkopen.2023.27421)
Supplement: Supplement 1. — eMethods. eTable 1. Baseline Characteristics of the Study Population by Tertiles of Neighborhood Socioeconomic Position During Midlife: The ARIC Study 1990-2019 (N=5538) eTable 2. Combined Estimates of Percentage Difference From Median Rate of Cognitive Decline Between the Ages of 50 and 90 Years by Neighborhood Socioeconomic Position During Childhood and Midlife: The ARIC Study 1990-2019 (N=5711) eTable 3. Estimates of the Odds of Belonging to a Specific Quintile of Cognitive Decline Between the Ages of 50 and 90 Years by Tertiles of Neighborhood Socioeconomic Position During Childhood or Midlife: The ARIC Study 1990-2019 (N=5711) eTable 4. Combined Estimates of the Odds of Belonging to a Specific Quintile of Cognitive Decline Between the Ages of 50 and 90 Years by Continuous, Standardized Measures of Neighborhood Socioeconomic Position During Childhood and Midlife: The ARIC Study 1990-2019 (N=5711) eTable 5. Combined Estimates of the Odds of Belonging to a Specific Quintile of Cognitive Decline Between the Ages of 50 and 90 Years by Tertiles of Neighborhood Socioeconomic Position During Childhood and Midlife: The ARIC Study 1990-2019 (N=5711) eTable 6. Stratified Estimates of Percentage Difference From Median Rate of Cognitive Decline Between the Ages of 50 and 90 Years by Continuous, Standardized Measures of Neighborhood Socioeconomic Position During Childhood or Midlife: The ARIC Study 1990-2019 (N=5711) eTable 7. Stratified Estimates of the Odds of Belonging to a Specific Quintile of Cognitive Decline Between the Ages of 50 and 90 Years by Continuous, Standardized Measures of Neighborhood Socioeconomic Position During Midlife: The ARIC Study 1990-2019 (N=5711) eFigure. Flowchart of Participants Selected for Analysis eReferences. [file jamanetwopen-e2327421-s001.pdf]

Supplementary Online Content

Kucharska-Newton AM, Pike JR, Chen J, et al. Association of childhood and midlife neighborhood socioeconomic position with cognitive decline. *JAMA Netw Open.* 2023;6(8):e2327421. doi:10.1001/jamanetworkopen.2023.27421

eMethods.
eTable 1. Baseline Characteristics of the Study Population by Tertiles of Neighborhood Socioeconomic Position During Midlife: The ARIC Study 1990-2019 (N=5538)
eTable 2. Combined Estimates of Percentage Difference From Median Rate of Cognitive Decline Between the Ages of 50 and 90 Years by Neighborhood Socioeconomic Position During Childhood and Midlife: The ARIC Study 1990-2019 (N=5711)
eTable 3. Estimates of the Odds of Belonging to a Specific Quintile of Cognitive Decline Between the Ages of 50 and 90 Years by Tertiles of Neighborhood Socioeconomic Position During Childhood or Midlife: The ARIC Study 1990-2019 (N=5711)
eTable 4. Combined Estimates of the Odds of Belonging to a Specific Quintile of Cognitive Decline Between the Ages of 50 and 90 Years by Continuous, Standardized Measures of Neighborhood Socioeconomic Position During Childhood and Midlife: The ARIC Study 1990-2019 (N=5711)
eTable 5. Combined Estimates of the Odds of Belonging to a Specific Quintile of Cognitive Decline Between the Ages of 50 and 90 Years by Tertiles of Neighborhood Socioeconomic Position During Childhood and Midlife: The ARIC Study 1990-2019 (N=5711)
eTable 6. Stratified Estimates of Percentage Difference From Median Rate of Cognitive Decline Between the Ages of 50 and 90 Years by Continuous, Standardized Measures of Neighborhood Socioeconomic Position During Childhood or Midlife: The ARIC Study 1990-2019 (N=5711)
eTable 7. Stratified Estimates of the Odds of Belonging to a Specific Quintile of Cognitive Decline Between the Ages of 50 and 90 Years by Continuous, Standardized Measures of Neighborhood Socioeconomic Position During Midlife: The ARIC Study 1990-2019 (N=5711)
eFigure. Flowchart of Participants Selected for Analysis
eReferences.

This supplementary material has been provided by the authors to give readers additional information about their work.

© 2023 Kucharska-Newton AM et al. *JAMA Network Open.*

eMethods.

Additional Measures Obtained at ARIC Visit 2 (1990-1992)

Plasma HDL-cholesterol levels were measured using the method of Warnick et al.[1] Plasma total cholesterol levels were determined enzymatically using a Cobas-Bio analyzer with reagents purchased from Boehringer Mannheim Biochemicals, Indianapolis, IN.[2] Plasma LDL-cholesterol levels were estimated using the Friedewald formula.[3] Body weight was measured to the nearest 0.1 kilogram and height was recorded to the nearest centimeter. Body mass index was calculated as the ratio of weight in kilograms to height in meters squared. Sitting blood pressure was measured three times using a random zero sphygmomanometer, with blood pressure estimated as an average of the second and third measurement. Hypertension was defined as present based on use of antihypertensive medication within two weeks of baseline data collection, systolic blood pressure greater than or equal to 140 mm Hg, or diastolic blood pressure greater than or equal to 90 mm Hg. Diabetes was defined as present based on a self-reported physician's diagnosis of diabetes, use of hypoglycemic medications, non-fasting serum glucose levels greater than or equal to 11.1 mmol/L, or fasting (≥ 8 hr) serum glucose level greater than or equal to 7.0 mmol/L. Multimorbidity at visit 2 (1990-1992) was defined as the presence of at least two of the following chronic conditions in an individual: cancer, diabetes, myocardial infarction, stroke, chronic obstructive pulmonary disease, heart failure, or hypertension.[4,5] Weekly physical activity (in minutes per week) was assessed using the interviewer-administered Modified Baecke questionnaire.[6] Participants indicated up to four sport activities performed within the last year, the frequency (hours/week) with which they performed each activity, and duration of activity (months/year). Each activity was assigned a value in minutes per week.[7] A sum from all four activities was computed. Participants who reported that they did not engage in any physical activity were assigned a value of 0 minutes per week.

Time-Varying Measures Utilized As Auxiliary Variables During Imputation

Time-varying measures obtained at visit 2 were repeated at subsequent visits and included in the imputation model. The model also incorporated additional measures obtained during phone-based assessments conducted annually since 1988 and semi-annually since 2012 or abstracted from medical records and death certificates. Self-reported use of alcohol (current, former, never) was assessed at each visit. Self-reported use of cigarettes (current, former, never) was ascertained at each visit and verified during follow-up phone calls. The Mini-Mental State Exam[8] was completed at visits 5, 6, and 7 and the Six-Item Screener (SIS)[9] was administered during visits 6 and 7. Self-rated health[10] was measured via phone. During the call, ARIC staff cataloged the number of hospitalizations since the last assessment and the use of a proxy during the assessment. Medical records and death certificates were systematically reviewed to identify incident heart failure, myocardial infarction, and stroke. Incident dementia was determined by adjudicated review, telephone interviews, informant interviews, hospitalization records, and death certificates.[11]

Imputation Process

The imputation model included all measures previously described. The number of years between visit 2 and each clinic-based follow-up assessment was calculated for each participant and integrated into the imputation model. If a participant did not attend a visit but completed the SIS, the time between visit 2 and the SIS was computed. If a participant did not attend a visit and had a known date of death, then the date 180 days beforehand was used. If none of these sources of information were available, then the time between the baseline and the median of all

© 2023 Kucharska-Newton AM et al. *JAMA Network Open.*

completed clinic-based follow-up assessments was utilized. The imputation model incorporated interactions between these time variables and the factor scores of cognitive function. Interactions were also specified between incident dementia and sex, race-center, education, and age at visit 2. The square of age at visit 2 was included to allow for a nonlinear relationship between age and cognitive decline. Pre-death cognitive scores were imputed from five separate sub-models. The first sub-model included all analytic and auxiliary measures from visit 2. The second sub-model included all measures from visits 2 and 4. The third sub-model added a dichotomous variable that indicated if the participant died before the visit 5 examination period concluded and used measures from visits 2, 4, and 5. The fourth sub-model was restricted to participants who were alive at the start of visit 6, included a dichotomous variable for participants who died before the end of visit 6, and integrated measures from visits 2, 4, 5, and 6. The final sub-model was restricted to participants alive at the start of visit 7, included a variable for participants who died before the end of visit 7, and used all measures from all visits. This iterative process ensured that concurrent and past variables, but not future variables, informed the values generated for missing cognitive factor scores among living participants.

© 2023 Kucharska-Newton AM et al. *JAMA Network Open.*

eTable 1. Baseline Characteristics of the Study Population by Tertiles of Neighborhood Socioeconomic Position During Midlife: The ARIC Study 1990-2019 (N=5538)

		Tertile of Midlife Neighborhood SEP			
	All (N=5538)	Low Z score range -12.84 to -0.98 (N=1869)	Intermediate Z score range -0.98 to 2.95 (N=1821)	High Z score range 2.97 to 14.78 (N=1848)	P Trend
Age					
No.	5538	1869	1821	1848	NA
Mean (SD), y	54.9 (4.6)	55.2 (4.8)	55.1 (4.5)	54.5 (4.4)	<.001
Decade of birth, No. (%)					
1920-1929	584/5538 (10.5)	253/1869 (13.5)	183/1821 (10.0)	148/1848 (8.0)	<.001
1930-1939	3309/5538 (59.8)	1081/1869 (57.8)	1128/1821 (61.9)	1100/1848 (59.5)	
1940-1949	1645/5538 (29.7)	535/1869 (28.6)	510/1821 (28.0)	600/1848 (32.5)	
Female sex, No. (%)	3267/5538 (59.0)	1204/1869 (64.4)	1002/1821 (55.0)	1061/1848 (57.4)	<.001
Black race, No. (%)	1275/5538 (23.0)	1098/1869 (58.7)	31/1821 (1.7)	146/1848 (7.9)	<.001
Race and center, No. (%)					
White, Forsyth County, North Carolina	1114/5538 (20.1)	197/1869 (10.5)	347/1821 (19.1)	570/1848 (30.8)	<.001
Black, Forsyth County, North Carolina	78/5538 (1.4)	69/1869 (3.7)	7/1821 (0.4)	2/1848 (0.1)	
White, Minneapolis, Minnesota	1684/5538 (30.4)	43/1869 (2.3)	667/1821 (36.6)	974/1848 (52.7)	
White, Washington County, Maryland	1465/5538 (26.5)	531/1869 (28.4)	776/1821 (42.6)	158/1848 (8.5)	
Black, Jackson, Mississippi	1197/5538 (21.6)	1029/1869 (55.1)	24/1821 (1.3)	144/1848 (7.8)	
Education, No. (%)					
Less than high school	788/5538 (14.2)	529/1869 (28.3)	201/1821 (11.0)	58/1848 (3.1)	<.001
High school, GED, or vocational school	2343/5538 (42.3)	758/1869 (40.6)	960/1821 (52.7)	625/1848 (33.8)	
College, graduate, or professional school	2407/5538 (43.5)	582/1869 (31.1)	660/1821 (36.2)	1165/1848 (63.0)	
One or more APOE ε4 alleles, No. (%)	1549/5358 (28.9)	610/1821 (33.5)	445/1746 (25.5)	494/1791 (27.6)	<.001
Systolic blood pressure					
No.	5509	1850	1812	1847	NA
Mean (SD), mm HG	117.4 (16.6)	120.5/1869 (18.0)	115.9 (15.3)	115.7 (15.9)	<.001
Hypertension, No. (%)	1716/5538 (31.0)	787 (42.1)	500/1821 (27.5)	429/1848 (23.2)	<.001
Body mass index					
No.	5507	1850	1810	1847	NA
Mean (SD)	27.7 (5.1)	29.0 (5.8)	27.4 (4.7)	26.7 (4.5)	<.001
Diabetes, No. (%)	478/5490 (8.7)	234/1835 (12.8)	128/1811 (7.1)	116/1844 (6.3)	<.001
Average sport mins/week					
No.	5535	1867	1821	1847	NA
Mean (SD), mins/wk	669.4 (811.4)	468.9 (688.3)	694.0 (805.6)	847.9 (884.2)	<.001
HDL cholesterol					
No.	5474	1834	1804	1836	NA
Mean (SD), mg/dL	50.9 (16.7)	51.4 (16.6)	48.8 (16.2)	52.6 (17.3)	<.001
LDL cholesterol					
No.	5406	1813	1778	1815	NA
Mean (SD), mg/dL	130.6 (35.0)	132.5 (36.7)	131.5 (34.3)	127.9 (33.8)	<.001
Multimorbidity, No. (%)	449/5538 (8.1)	225/1869 (12.0)	117/1821 (6.4)	107/1848 (5.8)	<.001
Childhood nSEP					
No.	5538	1869	1821	1848	NA
Mean (SD)	0.6 (4.0)	-0.7 (3.9)	1 (3.8)	1.6 (4.0)	<.001
Midlife nSEP					
No.	5538	1869	1821	1848	NA
Mean (SD)	1.1 (4.8)	-4.1 (2.5)	1 (0.9)	6.3 (2.8)	<.001
Global cognition, mean (SD)					
No.	5517	1854	1815	1848	NA
Mean (SD)	0.0 (1.0)	-0.6 (1.0)	0.2 (0.8)	0.4 (0.8)	<.001
Death by 2019, No. (%)	1250/5538 (22.6)	509/1869 (27.2)	408/1821 (22.4)	333/1848 (18.0)	<.001

Abbreviations: ARIC, Atherosclerosis Risk in Communities; APOE, apolipoprotein E; kg, kilogram; HDL, high-density lipoprotein; LDL, low-density lipoprotein; MET, metabolic equivalent of task; mg/dL, milligrams per deciliter; min, minute; mm HG, millimetre of mercury; wk, week.

Study baseline (1990-92) defined as the first year in which a participant in the Atherosclerosis Risk in Communities study completed a neuropsychological assessment. Univariate baseline differences in study variables were assessed using linear regression, Cochran-Armitage trend tests, Cochran-Mantel-Haenszel trend tests as appropriate.

© 2023 Kucharska-Newton AM et al. *JAMA Network Open.*

eTable 2. Combined[a] Estimates of Percentage Difference From Median Rate of Cognitive Decline Between the Ages of 50 and 90 Years by Neighborhood Socioeconomic Position During Childhood and Midlife: The ARIC Study 1990-2019 (N=5711)

	No. nSEP[b]	% (95% CI) Model 1	% (95% CI) Model 2
Childhood nSEP (Standardized)	5711	-15.0% (-17.3%, -12.6%)	-5.7% (-8.1%, -3.4%)
Midlife nSEP (Standardized)	5711	0.1% (-2.3%, 2.5%)	0.3% (-2.1%, 2.8%)
Tertiles of Childhood nSEP			
Low	1906	0% [Reference]	0% [Reference]
Intermediate	1901	-18.9% (-24.1%, -13.7%)	-7.3% (-12.3%, -2.2%)
High	1904	-30.4% (-35.9%, -24.9%)	-11.4% (-16.9%, -6.0%)
Tertiles of Midlife nSEP			
Low	1886	0% [Reference]	0% [Reference]
Intermediate	1917	-0.6% (-6.1%, 5.0%)	-0.0% (-5.4%, 5.3%)
High	1908	-0.9% (-6.6%, 4.7%)	0.1% (-5.6%, 5.8%)

Symbols and Abbreviations: [a]Indicates that both childhood and midlife nSEP were included in the same model; [b]Includes imputed values for midlife nSEP; ARIC, Atherosclerosis Risk in Communities; CI, confidence intervals; OR, odds ratio, nSEP, neighborhood socioeconomic position.

A two-stage process was employed to estimate the association between nSEP and cognitive decline. During the first stage, multivariate imputation by chained equations was employed to impute missing data. A linear mixed effects model that specified time from age 50 as the timescale, employed an unstructured variance-covariance matrix, and incorporated a random intercept and slope was fit to imputed data. Sex and race-center were included as covariates and an interaction between each covariate and time was added to the model. Random effects representing subject-specific rates of cognitive decline after age 50 were computed and converted into a percentage by dividing by the sample median. During the second stage, linear mixed effects models with random intercepts at the neighborhood-level estimated the association between nSEP and the percentage difference from the median rate of cognitive decline. Model 1 adjusted for sex and race-center. Model 2 additionally adjusted for birth decade, education, and APOE ε4.

eTable 3. Estimates of the Odds of Belonging to a Specific Quintile of Cognitive Decline Between the Ages of 50 and 90 Years by Tertiles of Neighborhood Socioeconomic Position During Childhood or Midlife: The ARIC Study 1990-2019 (N=5711)

	**No. nSEP / No. Per Quintile[a] (%)**	**OR (95% CI) Model 1**	**OR (95% CI) Model 2**
Least Amount of Cognitive Decline (5th Quintile) Compared to Median (3rd Quintile)			
Childhood nSEP (Low)	260/1142 (22.8%)	1 [Reference]	1 [Reference]
Childhood nSEP (Intermediate)	431/1142 (37.7%)	1.82 (1.44, 2.31)	1.63 (1.28, 2.08)
Childhood nSEP (High)	450/1142 (39.4%)	2.08 (1.60, 2.71)	1.61 (1.20, 2.17)
Midlife nSEP (Low)	382/1142 (33.5%)	1 [Reference]	1 [Reference]
Midlife nSEP (Intermediate)	378/1142 (33.1%)	0.87 (0.69, 1.11)	0.91 (0.69, 1.20)
Midlife nSEP (High)	382/1142 (33.5%)	0.82 (0.65, 1.03)	0.92 (0.66, 1.28)
Second Least Amount of Cognitive Decline (4th Quintile) Compared to Median (3rd Quintile)			
Childhood nSEP (Low)	323/1142 (28.3%)	1 [Reference]	1 [Reference]
Childhood nSEP (Intermediate)	376/1142 (32.9%)	1.23 (0.97, 1.54)	1.17 (0.91, 1.52)
Childhood nSEP (High)	443/1142 (38.8%)	1.30 (1.04, 1.63)	1.25 (0.94, 1.65)
Midlife nSEP (Low)	335/1142 (29.3%)	1 [Reference]	1 [Reference]
Midlife nSEP (Intermediate)	412/1142 (36.1%)	1.07 (0.83, 1.39)	1.16 (0.88, 1.52)
Midlife nSEP (High)	395/1142 (34.6%)	0.97 (0.74, 1.27)	1.00 (0.71, 1.41)
Second Greatest Amount of Cognitive Decline (2nd Quintile) Compared to Median (3rd Quintile)			
Childhood nSEP (Low)	430/1142 (37.7%)	1 [Reference]	1 [Reference]
Childhood nSEP (Intermediate)	373/1142 (32.7%)	0.88 (0.70, 1.12)	1.05 (0.82, 1.35)
Childhood nSEP (High)	339/1142 (29.7%)	0.59 (0.46, 0.77)	0.83 (0.61, 1.13)
Midlife nSEP (Low)	384/1142 (33.6%)	1 [Reference]	1 [Reference]
Midlife nSEP (Intermediate)	365/1142 (32.0%)	0.83 (0.65, 1.05)	0.87 (0.66, 1.15)
Midlife nSEP (High)	393/1142 (34.4%)	0.84 (0.65, 1.08)	0.94 (0.66, 1.32)
Greatest Amount of Cognitive Decline (1st Quintile) Compared to Median (3rd Quintile)			
Childhood nSEP (Low)	513/1142 (44.9%)	1 [Reference]	1 [Reference]
Childhood nSEP (Intermediate)	359/1142 (31.4%)	0.65 (0.52, 0.81)	0.94 (0.74, 1.19)
Childhood nSEP (High)	271/1142 (23.7%)	0.33 (0.25, 0.44)	0.70 (0.50, 0.99)
Midlife nSEP (Low)	444/1142 (38.9%)	1 [Reference]	1 [Reference]
Midlife nSEP (Intermediate)	372/1142 (32.6%)	0.73 (0.58, 0.92)	0.87 (0.67, 1.13)
Midlife nSEP (High)	326/1142 (28.5%)	0.60 (0.48, 0.76)	0.82 (0.60, 1.11)

Symbols and Abbreviations: [a]Includes imputed values for midlife nSEP; ARIC, Atherosclerosis Risk in Communities; CI, confidence intervals; OR, odds ratio, nSEP, neighborhood socioeconomic position.

A two-stage process was employed to estimate the association between nSEP and cognitive decline. During the first stage, multivariate imputation by chained equations was employed to impute missing data. A linear mixed effects model that specified time from age 50 as the timescale, employed an unstructured variance-covariance matrix, and incorporated a random intercept and slope was fit to imputed data. Sex and race-center were included as covariates and an interaction between age and each covariate was added to the model. Random effects representing subject-specific rates of cognitive decline after age 50 were computed and discretized into quintiles. During the second stage, multinomial logistic mixed effects models with random intercepts at the neighborhood-level estimated the odds of belonging to a specific quintile of cognitive decline. Separate models were fit for each measure of childhood and midlife nSEP. Model 1 adjusted for sex and race-center. Model 2 additionally adjusted for birth decade, education, and APOE ε4.

© 2023 Kucharska-Newton AM et al. *JAMA Network Open.*

eTable 4. Combined[a] Estimates of the Odds of Belonging to a Specific Quintile of Cognitive Decline Between the Ages of 50 and 90 Years by Continuous, Standardized Measures of Neighborhood Socioeconomic Position During Childhood and Midlife: The ARIC Study 1990-2019 (N=5711)

	No. Per Quintile[b]	OR (95% CI) Model 1	OR (95% CI) Model 2
Least Amount of Cognitive Decline (5th Quintile) Compared to Median (3rd Quintile)			
Childhood nSEP	1142	1.38 (1.23, 1.56)	1.18 (1.05, 1.34)
Midlife nSEP	1142	0.89 (0.80, 0.98)	0.89 (0.80, 0.99)
Second Least Amount of Cognitive Decline (4th Quintile) Compared to Median (3rd Quintile)			
Childhood nSEP	1142	1.13 (1.01, 1.25)	1.07 (0.96, 1.21)
Midlife nSEP	1142	0.97 (0.86, 1.10)	0.96 (0.83, 1.10)
Second Greatest Amount of Cognitive Decline (2nd Quintile) Compared to Median (3rd Quintile)			
Childhood nSEP	1142	0.78 (0.69, 0.89)	0.90 (0.78, 1.02)
Midlife nSEP	1142	0.99 (0.87, 1.11)	1.01 (0.88, 1.15)
Greatest Amount of Cognitive Decline (1st Quintile) Compared to Median (3rd Quintile)			
Childhood nSEP	1142	0.57 (0.50, 0.65)	0.79 (0.69, 0.92)
Midlife nSEP	1142	0.91 (0.82, 1.01)	0.92 (0.82, 1.04)

Symbols and Abbreviations: [a]Indicates that both childhood and midlife nSEP were included in the same model; [b]Includes imputed values for midlife nSEP; ARIC, Atherosclerosis Risk in Communities; CI, confidence intervals; OR, odds ratio, nSEP, neighborhood socioeconomic position.

A two-stage process was employed to estimate the association between nSEP and cognitive decline. During the first stage, multivariate imputation by chained equations was employed to impute missing data. A linear mixed effects model that specified time from age 50 as the timescale, employed an unstructured variance-covariance matrix, and incorporated a random intercept and slope was fit to imputed data. Sex and race-center were included as covariates and an interaction between each covariate and time was added to the model. Random effects representing subject-specific rates of cognitive decline after age 50 were computed and discretized into quintiles. During the second stage, multinomial logistic mixed effects models with random intercepts at the neighborhood-level estimated the odds of belonging to a specific quintile of cognitive decline. Model 1 adjusted for sex and race-center. Model 2 additionally adjusted for birth decade, education, and APOE ε4.

© 2023 Kucharska-Newton AM et al. *JAMA Network Open.*

eTable 5. Combined[a] Estimates of the Odds of Belonging to a Specific Quintile of Cognitive Decline Between the Ages of 50 and 90 Years by Tertiles of Neighborhood Socioeconomic Position During Childhood and Midlife: The ARIC Study 1990-2019 (N=5711)

	No. nSEP / No. Per Quintile[b] (%)	OR (95% CI) Model 1	OR (95% CI) Model 2
Least Amount of Cognitive Decline (5th Quintile) Compared to Median (3rd Quintile)			
Tertiles of Childhood nSEP			
Low	260/1142 (22.8%)	1 [Reference]	1 [Reference]
Intermediate	431/1142 (37.7%)	1.88 (1.48, 2.39)	1.56 (1.22, 2.00)
High	450/1142 (39.4%)	2.18 (1.66, 2.86)	1.59 (1.18, 2.15)
Tertiles of Midlife nSEP			
Low	382/1142 (33.5%)	1 [Reference]	1 [Reference]
Intermediate	378/1142 (33.1%)	0.81 (0.63, 1.03)	0.83 (0.64, 1.06)
High	382/1142 (33.5%)	0.77 (0.60, 0.98)	0.78 (0.61, 1.00)
Second Least Amount of Cognitive Decline (4th Quintile) Compared to Median (3rd Quintile)			
Tertiles of Childhood nSEP			
Low	323/1142 (28.3%)	1 [Reference]	1 [Reference]
Intermediate	376/1142 (32.9%)	1.23 (0.97, 1.57)	1.15 (0.89, 1.49)
High	443/1142 (38.8%)	1.33 (1.04, 1.70)	1.21 (0.92, 1.59)
Tertiles of Midlife nSEP			
Low	335/1142 (29.3%)	1 [Reference]	1 [Reference]
Intermediate	412/1142 (36.1%)	1.03 (0.79, 1.35)	1.02 (0.77, 1.34)
High	395/1142 (34.6%)	0.91 (0.68, 1.22)	0.88 (0.64, 1.21)
Second Greatest Amount of Cognitive Decline (2nd Quintile) Compared to Median (3rd Quintile)			
Tertiles of Childhood nSEP			
Low	430/1142 (37.7%)	1 [Reference]	1 [Reference]
Intermediate	373/1142 (32.7%)	0.90 (0.70, 1.14)	1.03 (0.81, 1.33)
High	339/1142 (29.7%)	0.61 (0.46, 0.80)	0.81 (0.60, 1.10)
Tertiles of Midlife nSEP			
Low	384/1142 (33.6%)	1 [Reference]	1 [Reference]
Intermediate	365/1142 (32.0%)	0.90 (0.70, 1.17)	0.93 (0.71, 1.20)
High	393/1142 (34.4%)	0.93 (0.70, 1.23)	1.00 (0.74, 1.34)
Greatest Amount of Cognitive Decline (1st Quintile) Compared to Median (3rd Quintile)			
Tertiles of Childhood nSEP			
Low	513/1142 (44.9%)	1 [Reference]	1 [Reference]
Intermediate	359/1142 (31.4%)	0.68 (0.54, 0.85)	0.92 (0.73, 1.17)
High	271/1142 (23.7%)	0.35 (0.26, 0.48)	0.70 (0.50, 0.98)
Tertiles of Midlife nSEP			
Low	444/1142 (38.9%)	1 [Reference]	1 [Reference]
Intermediate	372/1142 (32.6%)	0.83 (0.65, 1.06)	0.86 (0.66, 1.11)
High	326/1142 (28.5%)	0.71 (0.55, 0.92)	0.78 (0.59, 1.02)

Symbols and Abbreviations: [a]Indicates that both childhood and midlife nSEP were included in the same model; [b]Includes imputed values for midlife nSEP; ARIC, Atherosclerosis Risk in Communities; CI, confidence intervals; OR, odds ratio, nSEP, neighborhood socioeconomic position.

A two-stage process was employed to estimate the association between nSEP and cognitive decline. During the first stage, multivariate imputation by chained equations was employed to impute missing data. A linear mixed effects model that specified time from age 50 as the timescale, employed an unstructured variance-covariance matrix, and incorporated a random intercept and slope was fit to imputed data. Sex and race-center were included as covariates and an interaction between each covariate and time was added to the model. Random effects representing subject-specific rates of cognitive decline after age 50 were computed and discretized into quintiles. During the second stage, multinomial logistic mixed effects models with random intercepts at the neighborhood-level estimated the odds of belonging to a specific quintile of cognitive decline. Model 1 adjusted for sex and race-center. Model 2 additionally adjusted for birth decade, education, and APOE ε4.

© 2023 Kucharska-Newton AM et al. *JAMA Network Open.*

eTable 6. Stratified Estimates of Percentage Difference From Median Rate of Cognitive Decline Between the Ages of 50 and 90 Years by Continuous, Standardized Measures of Neighborhood Socioeconomic Position During Childhood or Midlife: The ARIC Study 1990-2019 (N=5711)

	No. Per Subgroup	% (95% CI) Childhood nSEP	% (95% CI) Midlife nSEP
**Race**			
Black	1313	-6.4% (-13.0%, 0.2%)	-0.8% (-6.8%, 5.3%)
White	4398	-11.2% (-14.5%, -7.9%)	-1.5% (-4.4%, 1.3%)
**Decade of Birth**			
1920-1929	687	-3.2% (-14.0%, 7.5%)	-8.6% (-19.0%, 1.7%)
1930-1939	3355	-11.6% (-15.1%, -8.2%)	0.5% (-4.2%, 5.3%)
1940-1949	1669	-1.8% (-5.6%, 2.0%)	1.6% (-3.1%, 6.2%)*
**Sex**			
Female	3372	-10.6% (-14.4%, -6.7%)	-2.0% (-5.2%, 1.1%)
Male	2339	-5.7% (-9.2%, -2.1%)	1.0% (-2.3%, 4.2%)
**Multimorbidity**			
2+ conditions	473	-2.5% (-10.4%, 5.4%)	-2.2% (-10.8%, 6.4%)
0 or 1 conditions	5238	-9.2% (-12.1%, -6.3%)	-0.4% (-2.6%, 1.8%)

Symbols and Abbreviations: *additive interaction P <.05; ARIC, Atherosclerosis Risk in Communities; CI, confidence intervals; nSEP, neighborhood socioeconomic position.

A two-stage process was employed to estimate subject-specific cognitive decline between the ages of 50 and 90 years. Linear mixed effects model quantified the percentage difference from the median rate of cognitive decline. P-values for interactions were computed by specifying a cross-level interaction between neighborhood-level socioeconomic position and a person-level covariate. Effect modification was evaluated by stratifying the dataset by a select covariate. All models adjusted for sex, race-center, birth decade, education, and APOE ε4. Stratified models did not adjust for the covariate used to subset the data.

© 2023 Kucharska-Newton AM et al. *JAMA Network Open.*

eTable 7. Stratified Estimates of the Odds of Belonging to a Specific Quintile of Cognitive Decline Between the Ages of 50 and 90 Years by Continuous, Standardized Measures of Neighborhood Socioeconomic Position During Midlife: The ARIC Study 1990-2019 (N=5711)

	No. Per Subgroup	OR (95% CI) Least Amount of Cognitive Decline 5th Quintile to 3rd Quintile	OR (95% CI) Second Least Amount of Cognitive Decline 4th Quintile to 3rd Quintile	OR (95% CI) Second Greatest Amount of Cognitive Decline 2nd Quintile to 3rd Quintile	OR (95% CI) Greatest Amount of Cognitive Decline 1st Quintile to 3rd Quintile
**Race**					
Black	1313	1.32 (0.93, 1.87)	1.07 (0.71, 1.61)	1.03 (0.71, 1.50)	1.12 (0.78, 1.60)
White	4398	0.95 (0.77, 1.17)	0.96 (0.78, 1.18)	0.99 (0.80, 1.22)	0.94 (0.77, 1.15)
**Decade of Birth**					
1920-1929	687	0.74 (0.41, 1.33)	0.85 (0.51, 1.41)	0.78 (0.52, 1.18)	0.68 (0.49, 0.93)
1930-1939	3355	1.02 (0.82, 1.25)	1.05 (0.83, 1.31)	0.97 (0.79, 1.19)	0.96 (0.78, 1.17)
1940-1949	1669	1.01 (0.79, 1.30)	1.06 (0.80, 1.41)	0.99 (0.74, 1.32)	1.20 (0.89, 1.64)
**Sex**					
Female	3372	1.02 (0.85, 1.23)	1.05 (0.84, 1.32)	0.94 (0.77, 1.14)	0.94 (0.77, 1.15)
Male	2339	0.89 (0.70, 1.13)	0.96 (0.77, 1.21)	0.98 (0.78, 1.23)	0.92 (0.73, 1.16)
**Multimorbidity**					
2+ conditions	473	1.29 (0.71, 2.35)	1.67 (0.89, 3.17)	1.23 (0.72, 2.10)	1.29 (0.73, 2.29)
0 or 1 conditions	5238	0.94 (0.80, 1.10)	0.99 (0.84, 1.18)	0.95 (0.79, 1.13)	0.92 (0.78, 1.07)

Abbreviations: ARIC, Atherosclerosis Risk in Communities; CI, confidence intervals; OR, odds ratio.

A two-stage process was employed to estimate subject-specific cognitive decline between the ages of 50 and 90 years. Multinomial logistic mixed effects model estimated the odds of belonging to a specific quintile of cognitive decline. P-values for additive interactions were computed by calculating the relative excess risk due to interaction. P-values for multiplicative interactions were computed by specifying a cross-level interaction between neighborhood-level socioeconomic position and a person-level covariate. Effect modification was evaluated by stratifying the dataset by a select covariate. All models adjusted for sex, race-center, birth decade, education, and APOE ε4. Stratified models did not adjust for the covariate used to subset the data.

© 2023 Kucharska-Newton AM et al. *JAMA Network Open.*

eFigure. Flowchart of Participants Selected for Analysis

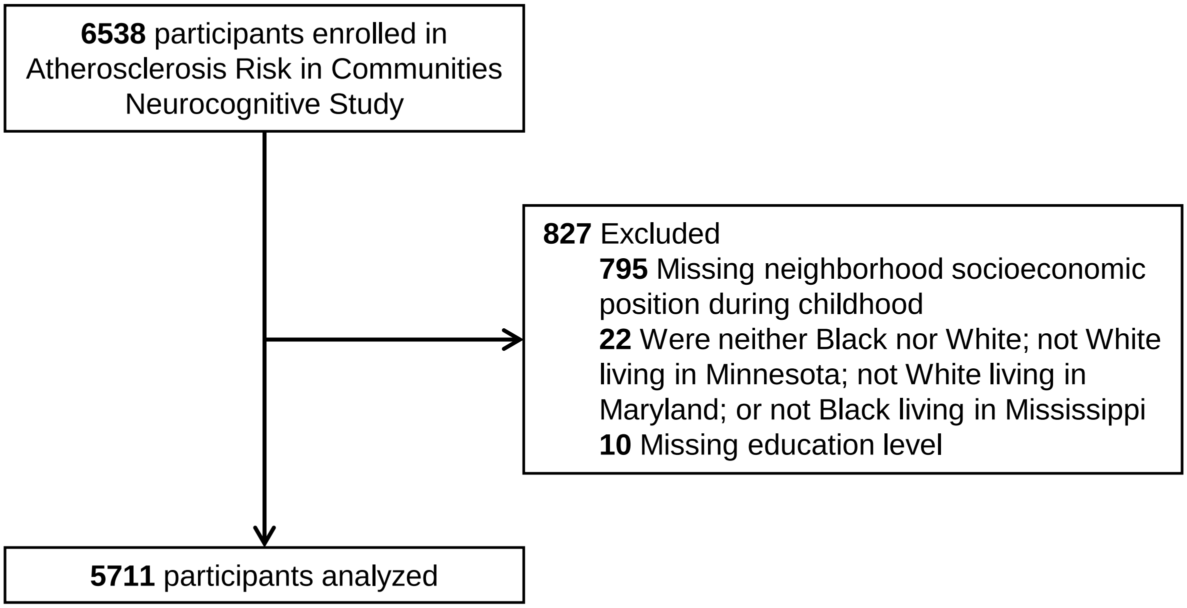

© 2023 Kucharska-Newton AM et al. *JAMA Network Open.*

eReferences.

1. Warnick GR, Mayfield C, Benderson J, Chen JS, Albers JJ. HDL cholesterol quantitation by phosphotungstate-Mg2+ and by dextran sulfate-Mn2+-polyethylene glycol precipitation, both with enzymic cholesterol assay compared with the lipid research method. *Am J Clin Pathol*. Nov 1982;78(5):718-23. doi:10.1093/ajcp/78.5.718
2. Siedel J, Hagele EO, Ziegenhorn J, Wahlefeld AW. Reagent for the enzymatic determination of serum total cholesterol with improved lipolytic efficiency. *Clin Chem*. Jun 1983;29(6):1075-80.
3. Friedewald WT, Levy RI, Fredrickson DS. Estimation of the concentration of low-density lipoprotein cholesterol in plasma, without use of the preparative ultracentrifuge. *Clin Chem*. Jun 1972;18(6):499-502.
4. Griffith LE, Gilsing A, Mangin D, et al. Multimorbidity Frameworks Impact Prevalence and Relationships with Patient-Important Outcomes. *J Am Geriatr Soc*. Aug 2019;67(8):1632-1640. doi:10.1111/jgs.15921
5. Le Reste JY, Nabbe P, Manceau B, et al. The European General Practice Research Network presents a comprehensive definition of multimorbidity in family medicine and long term care, following a systematic review of relevant literature. *J Am Med Dir Assoc*. May 2013;14(5):319-25. doi:10.1016/j.jamda.2013.01.001
6. Baecke JA, Burema J, Frijters JE. A short questionnaire for the measurement of habitual physical activity in epidemiological studies. *Am J Clin Nutr*. Nov 1982;36(5):936-42. doi:10.1093/ajcn/36.5.936
7. Ainsworth BE, Haskell WL, Whitt MC, et al. Compendium of physical activities: an update of activity codes and MET intensities. *Med Sci Sports Exerc*. Sep 2000;32(9 Suppl):S498-504. doi:10.1097/00005768-200009001-00009
8. Folstein MF, Folstein SE, McHugh PR. "Mini-mental state". A practical method for grading the cognitive state of patients for the clinician. *J Psychiatr Res*. Nov 1975;12(3):189-98. doi:10.1016/0022-3956(75)90026-6
9. Callahan CM, Unverzagt FW, Hui SL, Perkins AJ, Hendrie HC. Six-item screener to identify cognitive impairment among potential subjects for clinical research. *Med Care*. Sep 2002;40(9):771-81. doi:10.1097/00005650-200209000-00007
10. Stewart AL, Hays RD, Ware JE, Jr. The MOS short-form general health survey. Reliability and validity in a patient population. *Med Care*. Jul 1988;26(7):724-35. doi:10.1097/00005650-198807000-00007
11. Knopman DS, Gottesman RF, Sharrett AR, et al. Mild Cognitive Impairment and Dementia Prevalence: The Atherosclerosis Risk in Communities Neurocognitive Study (ARIC-NCS). *Alzheimers Dement (Amst)*. 2016;2:1-11. doi:10.1016/j.dadm.2015.12.002

© 2023 Kucharska-Newton AM et al. *JAMA Network Open.*
