# Supplementary material for: Association of Childhood and Midlife Neighborhood Socioeconomic Position With Cognitive Decline
Source: JAMA Netw Open. 2023 Aug 4;6(8):e2327421. doi: 10.1001/jamanetworkopen.2023.27421 (PMC10403777; doi:10.1001/jamanetworkopen.2023.27421)
Supplement: Supplement 2. — Data Sharing Statement [file jamanetwopen-e2327421-s002.pdf]

Data Sharing Statement

Kucharska-Newton. Association of Childhood and Midlife Neighborhood Socioeconomic Position With Cognitive Decline. *JAMA Netw Open*. Published August 04, 2023. doi:10.1001/jamanetworkopen.2023.27421

Data
Data available: Yes
Data types: Deidentified participant data, Data dictionary
How to access data: ARIC study data are available through the BioLINCC database https://biolincc.nhlbi.nih.gov/home/
When available: With publication

Supporting Documents
Document types: None

Additional Information
Who can access the data: BioLINCC data are available upon reasonable request
Types of analyses: BioLINCC data are available for any analyses
Mechanisms of data availability: BioLINCC are available following completion of NHLBI Research Materials Distribution Agreement
